# The Muscle Carnosine Response to Beta-Alanine Supplementation: A Systematic Review With Bayesian Individual and Aggregate Data E-Max Model and Meta-Analysis

**DOI:** 10.3389/fphys.2020.00913

**Published:** 2020-08-14

**Authors:** Nathália Saffioti Rezende, Paul Swinton, Luana Farias de Oliveira, Rafael Pires da Silva, Vinicius da Eira Silva, Kleiner Nemezio, Guilherme Yamaguchi, Guilherme Giannini Artioli, Bruno Gualano, Bryan Saunders, Eimear Dolan

**Affiliations:** ^1^Applied Physiology and Nutrition Research Group, School of Physical Education and Sport, Rheumatology Division, Faculdade de Medicina FMUSP, Universidade de São Paulo, São Paulo, Brazil; ^2^School of Health Sciences, Robert Gordon University, Aberdeen, United Kingdom; ^3^Food Research Center, University of São Paulo, São Paulo, Brazil

**Keywords:** nutrition, physiology, metabolism, supplement, meta-analysis, histidine containing dipeptides, buffering, dosing

## Abstract

Beta-alanine (BA) supplementation increases muscle carnosine content (MCarn), and has many proven, and purported, ergogenic, and therapeutic benefits. Currently, many questions on the nature of the MCarn response to supplementation are open, and the response to these has considerable potential to enhance the efficacy and application of this supplementation strategy. To address these questions, we conducted a systematic review with Bayesian-based meta-analysis of all published aggregate data using a dose response (Emax) model. Meta-regression was used to consider the influence of potential moderators (including dose, sex, age, baseline MCarn, and analysis method used) on the primary outcome. The protocol was designed according to PRISMA guidelines and a three-step screening strategy was undertaken to identify studies that measured the MCarn response to BA supplementation. Additionally, we conducted an original analysis of all available individual data on the MCarn response to BA supplementation from studies conducted within our lab (*n* = 99). The Emax model indicated that human skeletal muscle has large capacity for non-linear MCarn accumulation, and that commonly used BA supplementation protocols may not come close to saturating muscle carnosine content. Neither baseline values, nor sex, appeared to influence subsequent response to supplementation. Analysis of individual data indicated that MCarn is relatively stable in the absence of intervention, and effectually all participants respond to BA supplementation (99.3% response [95%CrI: 96.2–100]).

## Introduction

Beta-alanine (BA) supplementation is a widely used dietary strategy, due to its proven efficacy in increasing skeletal muscle carnosine content (MCarn) (Harris et al., 2006). Carnosine is a dipeptide formed from the amino acids beta-alanine (BA) and L-histidine, and is present in high concentrations in human skeletal muscle (~20–30 mmol·kg^−1^ dry muscle). Its purported roles include: proton buffering (Dolan et al., 2018), anti-oxidation (Boldyrev et al., 2010), anti-glycation (Ghodsi and Kheirouri, 2018), metal chelation (Boldyrev et al., 2013), and influencing calcium sensitivity (Dutka and Lamb, 2004), and hence muscle contractility. These diverse physiological properties allow carnosine to contribute to multiple processes in skeletal muscle metabolism, and considerable research efforts have been made to investigate both means to increase it, and in what situations such increases are beneficial. BA availability is the limiting factor in intramuscular carnosine synthesis (Harris et al., 2006) and it is widely recognized that supplementation with this amino acid substantially increases MCarn. This supplementation strategy has proven efficacious in many situations, with the majority of research focusing on its ergogenic properties (Hill et al., 2007). A strong body of literature attests to the ability of BA supplementation to improve high-intensity exercise performance, with meta-analytic data indicating that it exerts its greatest ergogenic influence in capacity-based exercise tests that last between 30 s and 10 min (Saunders et al., 2017a). This ergogenic effect likely occurs due to MCarn's buffering action (Boldyrev et al., 2013; Dolan et al., 2019a), and has earned BA its place as one of the world's most popular, scientifically-backed and widely endorsed sports supplements available (Maughan et al., 2018). The therapeutic efficacy of BA supplementation is less well-investigated, although it appears to be a promising strategy, given its ability to increase MCarn, which in itself, may, potentially, have numerous therapeutic applications (Artioli et al., 2018), including roles in anti-senescence (Boldyrev et al., 2010), neuroprotection (De Marchis et al., 2000; Dobrota et al., 2005), tumor growth attenuation (Renner et al., 2010), improved clinical outcomes in participants with Parkinson's disease (Boldyrev et al., 2008) and enhanced glucose sensitivity (de Courten et al., 2016). It is important to highlight, that many of these purported benefits are based on animal, or *in vitro*, models, and it is yet to be determined whether BA supplementation can impact these processes and conditions. As such, further research is warranted to confirm the therapeutic or clinical efficacy of BA supplementation.

These wide-ranging proven, or purported, benefits of BA supplementation have created an ever-increasing market, and it is a very commonly used dietary supplement. But many questions remain open about the MCarn response to BA supplementation, and these questions must be addressed in order to optimize the efficacy and applicability of this nutritional strategy (Perim et al., 2019). It seems that substantial amounts of BA are required to increase MCarn, with most studies using doses of ~3.2–6.4 g·day^−1^, for periods ranging from 4 to 24 weeks. It is likely that these large amounts of BA are required because the incorporation of ingested BA into the muscle is very low, with ~3–6% of ingested BA estimated to contribute toward MCarn accumulation (Stegen et al., 2013; Blancquaert et al., 2015). BA uptake into the muscle seems to be rapid and efficient, however the ability of carnosine synthase to incorporate it into MCarn is far slower (Bakardjiev and Bauer, 1994; de Souza Goncalves et al., 2020) and so the remaining BA is likely to be converted toward other processes such as transamination or oxidation (Blancquaert et al., 2016). Despite this inefficiency in the use of supplemental BA to synthesize MCarn, very large increases, to the order of 200%, have been reported (Saunders et al., 2017b). The capacity of the muscle to uptake and increase MCarn, and the quantity of BA required to achieve saturation is not, however, currently known. Inter-individual variability in response to supplementation seems to be high (Saunders et al., 2017b), yet little is known about what factors underpin this variation, nor what the individual proportion of response to BA supplementation actually is. Factors such as age, sex, and baseline carnosine content may all theoretically impact subsequent response to supplementation, although consideration of individual datasets indicates that this may not be the case (Baguet et al., 2012; Stellingwerff et al., 2012). To address these, and other, questions, we conducted a comprehensive analysis, comprising various modeling techniques, to synthesize existing understanding about the nature of the MCarn response to BA supplementation. We also analyzed individual participant data from studies conducted within our laboratory; and considered these findings within the context of published summary data, which was analyzed using a frequently used dose-response model (Emax). All analyses were conducted from a Bayesian perspective, the advantages of which is that it allowed the flexibility to use a range of models in order to more fully represent the available data, while simultaneously allowing the results to be interpreted intuitively and probabilistically (Dunson, 2001).

## Materials and Methods

The protocol for this study was designed according to the Preferred Reporting Items for Systematic Reviews and Meta-Analysis (PRISMA) guidelines. The Population, Intervention, Comparator, Outcomes and Study Design (PICOS) approach was used to guide the inclusion and exclusion of studies for this review and are described in Table 1. In addition to conducting a systematic search of available literature, we also combined all available individual data from studies conducted within the authors lab, all of which used a dosing strategy of 6.4 g·day^−1^, for periods varying between 4 and 24 weeks. Muscle carnosine content was measured using HPLC analysis of muscle biopsy data, and the full protocol for this analysis is described elsewhere (Saunders et al., 2017b).

**Table 1:** Study inclusion and exclusion criteria.

Population	Healthy individuals of any age or physical activity level.
Intervention	Original studies investigating the effects of oral BA supplementation on skeletal MCarn content.
Comparator	No human comparators were required in the studies included in this review, although the data from placebo groups were used to quantify biological variability across the time periods investigated, when available.
Outcomes	The primary outcome was the effect of BA supplementation on skeletal MCarn concentration. Potential moderators to this response included dose, sex, age, baseline MCarn, and the method used to measure MCarn.
Study design	Controlled or uncontrolled intervention studies.

### Search Strategy

The search strategy was based on a three-step screening (title/abstract screening, full-text screen and full text appraisal), independently undertaken by two reviewers. This search was originally conducted to inform a systematic risk assessment on the use of BA supplementation (Dolan et al., 2019b). This risk assessment included all BA supplementation studies (including both human and animal models). One hundred and one human studies were included in that investigation and were subsequently screened to identify those that included an MCarn measurement. The protocol for that review was prospectively registered (PROSPERO registration no. CRD42017071843).

### Data Analysis

The present study comprised both individual and aggregate data meta-analyses from a Bayesian perspective. Individual data were pooled using mixed effects multilevel models. Analyses were performed on the outcome variable MCarn (absolute value) to quantify the effects of BA supplementation and random noise due to biological variation and measurement error. Additionally, proportion of response was estimated across controlled studies by calculating inter-individual difference in response to supplementation and comparing this to a non-zero increase in MCarn (Swinton et al., 2018). Bayesian estimates of the standard deviation in observed change from active and placebo groups were used to obtain the intervention response standard deviation (σ^∧^,_IR) describing inter-individual difference in response. Aggregate data meta-analyses were performed using published pre- and post-intervention mean and standard deviation values. Values were transformed into standardized mean differences (SMD) and sampling variance calculated using methods described previously (Saunders et al., 2017a). Three-level mixed effects models were used to quantify the effects of supplementation dose. Insufficient data were available to allow investigation of the interaction between daily dose and intervention duration and so the total cumulative dose ingested was selected as the primary outcome, which previous research has identified as being more influential than either daily dose or intervention duration (Stellingwerff et al., 2012; Church et al., 2017; Dolan et al., 2019b). Subset analyses using study covariates were used to assess the effects of sex, age, or measurement method on the main effect of BA supplementation. Finally, a model-based approach was employed to investigate the dose-response relationship between cumulative BA supplementation and the SMD. A standard four parameter sigmoid predicted maximum effect (Emax) model was estimated with:

$$\begin{array}{l} E=E_{0}+\frac{E_{\max}\times C^{\gamma }}{EC_{50}^{\gamma }+C^{\gamma }} \end{array}$$

Where *E* is the effect size (SMD), E_0 is the baseline effect, E_max is the maximum effect, EC_50 is the cumulative dose that provides 50% of the maximum effect, *C* is the input (cumulative dose) and γ is the Hill coefficient controlling the slope of the sigmoid response. Inferences from all models were performed on posterior samples generated by Markov Chain Monte Carlo with Bayesian 95% credible intervals (CrIs) constructed to enable probabilistic interpretations of parameter values. Models were run in OpenBUGS (version 3.2.3, MRC Biostatistics Unit) and in R (version 3.3.1 R Development Core Team) using the R2OpenBugs package.

## Results

### Group Study Characteristics

**Figure 1:**
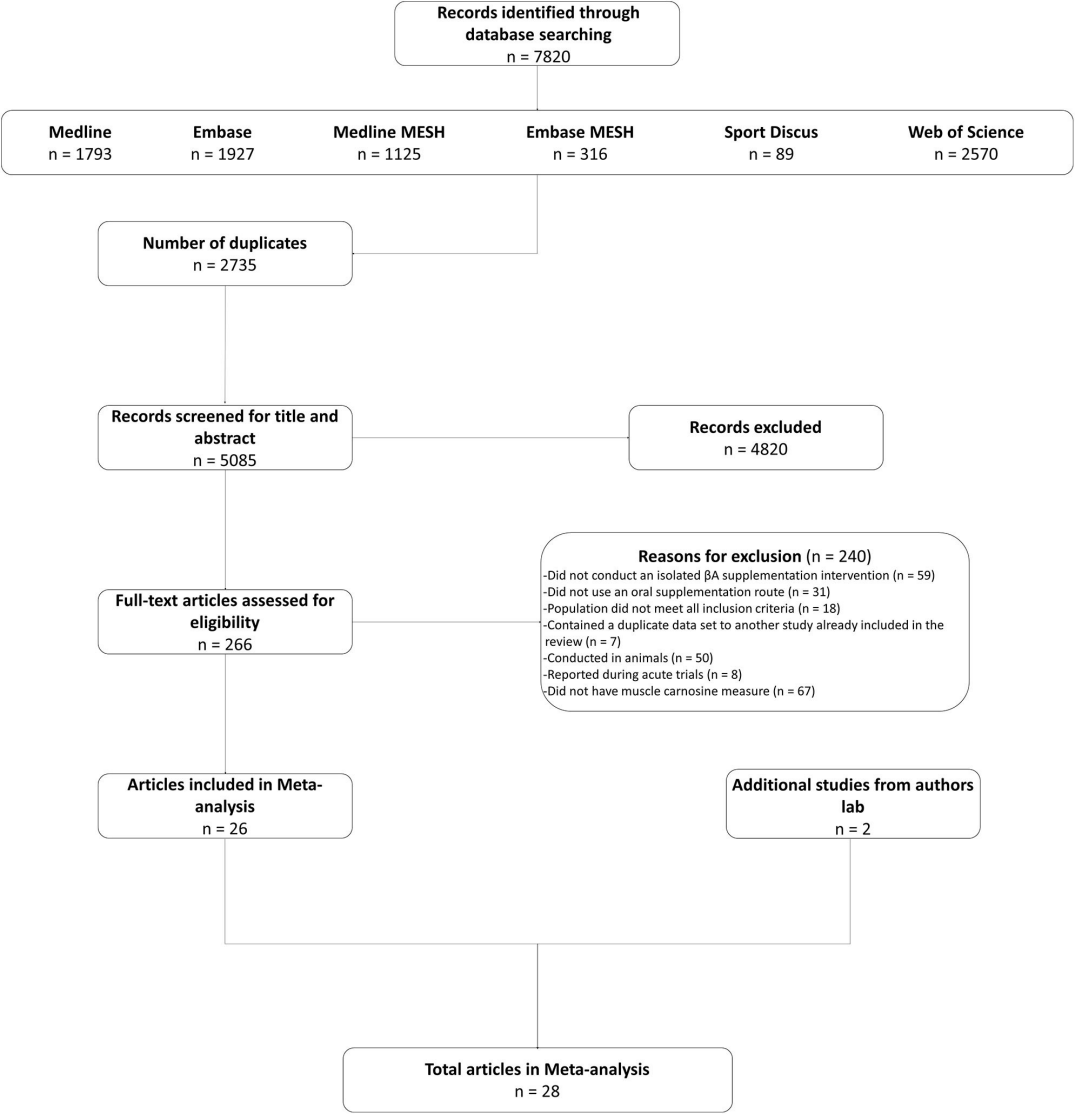
Search flow diagram.

Twenty-six studies were identified in the systematic search and included in the meta-analysis (Harris et al., 2006, 2010; Derave et al., 2007; Hill et al., 2007; Kendrick et al., 2008, 2009; Baguet et al., 2009, 2010; del Favero et al., 2012; Stellingwerff et al., 2012; Stegen et al., 2013; Bex et al., 2014, 2015; Chung et al., 2014; Danaher et al., 2014; Gross et al., 2014; Kresta et al., 2014; Cochran et al., 2015; Blancquaert et al., 2017; Church et al., 2017; Saunders et al., 2017b; Varanoske et al., 2017, 2018; Black et al., 2018; Carvalho et al., 2018; da Eira Silva et al., 2020). We also included data from two other, currently unpublished, studies conducted within the authors lab. These studies met all of the inclusion criteria described herein. The decision to include them was based on the additional power that the increased sample brought, as well as to ensure that the statistical analysis generated the best possible estimate of the true value (see Figure 1 for search flow diagram). In total, 575 participants (comprising 486 men and 89 women) were included in the meta-analysis, of which 382 consumed BA, with the remaining 193 allocated to a placebo intervention. The majority of studies were conducted on healthy young adults (mean age (yrs) = 23.89, *SD* = 5.46), with only one study conducted on older adults [mean age (yrs) = 64.34, *SD* = 4.99 (del Favero et al., 2012)] and none on younger adults (<18 years). An overview of all included studies is presented in Supplemental Table 1. Analyses were completed on subsets of the data depending on the specific analysis and suitability of each study set, as described below.

### Individual Data

Complete individual data sets were available for 99 participants (BAn = 67, PLAn = 32) comprising a total of 232 observations, some of which was previously published (Saunders et al., 2017b; Carvalho et al., 2018; da Eira Silva et al., 2020). All studies were conducted on young men and provided a BA dose of 6.4 g·day^−1^ with observations ranging from 4 to 24 weeks post baseline. BA supplementation increased MCarn on average by 16.0 mmol·kgDM^−1^ [95%CrI: 12.4–19.6] compared to placebo. Regression analyses with duration centered at 4 weeks were completed to determine if the effects of supplementation increased beyond this point (BAn = 50, 134 total observations). The mean change in MCarn at 4 weeks was 14.0 mmol·kgDM^−1^ [95%CrI: 10.1–18.1], with a positive regression slope indicating a further 0.5 [95%CrI: 0.2–0.7] mmol·kgDM^−1^ increase per week. Analyses of the same data also demonstrated that baseline levels of MCarn were not associated with changes due to supplementation (−0.1 [95%CrI: −0.3–0.1]). The amount of random noise in MCarn values due to biological variation and measurement error (i.e., typical variation) was estimated using observations from placebo groups. The standard deviation of residuals from the multilevel model representing typical variation was 4.1 mmol·kgDM^−1^ ([95%CrI: 3.4–5.1], PLAn = 18, 61 total observations). The intervention response standard deviation (σ^∧^,_IR) was estimated as 6.6 mmol·kgDM^−1^ [95%CrI: 3.4–9.4] and the proportion of individual response was 99.3% [95%CrI: 96.2–100].

### Aggregate Data

**Figure 2:**
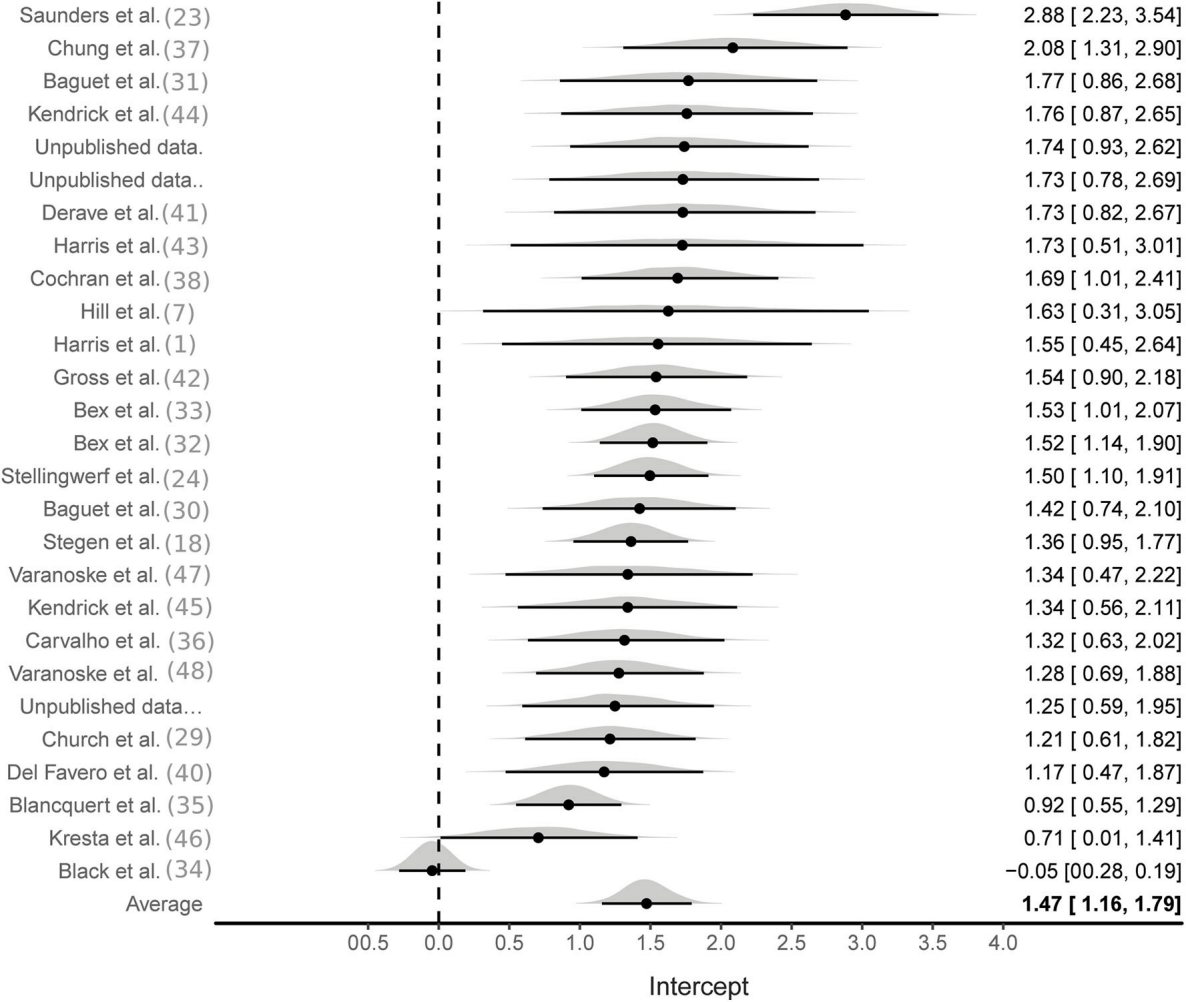
Bayesian forest plot of multilevel meta-analysis with non-controlled effect sizes.

**Figure 3:**
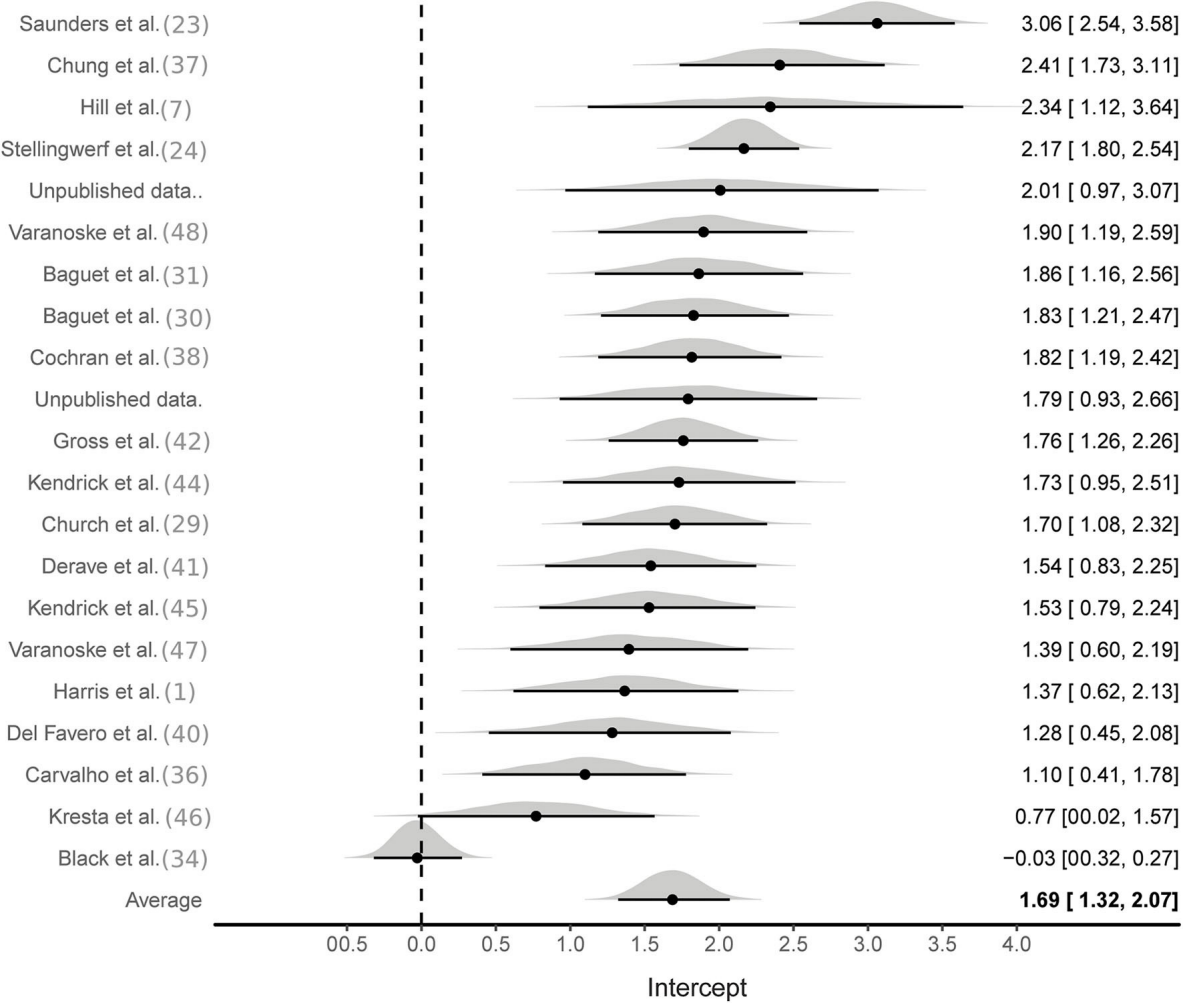
Bayesian forest plot of multilevel meta-analysis with controlled effect sizes.

Aggregate analyses were based on effect sizes calculated from all available studies using the SMD pre to post change in MCarn levels. One hundred and eight effect sizes were available from BA groups only, six of which were removed as they were outliers (ES > 5). The multilevel meta-analysis with no study covariates estimated a large pooled effect size of 1.5 [95%CrI 1.2–1.8], with substantial between (τ^∧^20.5 = 0.6) and within (ϵ^∧^20.5 = 0.7) study variance (Figure 2). The same model applied to effect sizes calculated with supplementation and control group data (22 studies and 56 effect sizes) also produced a large pooled effect size of 1.7 [95%CrI: 1.3–2.1], with substantial between (τ^∧^20.5 = 0.8) and within (ϵ^∧^20.5 = 0.5) study variance (Figure 3). Using a simple linear model, the effects of cumulative BA dose was assessed by centering on the mean value (208 g). Results demonstrated a large effect at the mean cumulative dose (1.5 [95%CrI: 1.2–1.8]) and an estimated 0.23 [95%CrI: 0.06–0.49] increase in effect size per additional 100 g. Similar results were obtained for effect sizes calculated with supplementation and control group data (effect at mean: 1.7 [95%CrI: 1.3–2.1]; effect per additional 100 g: 0.16 [95%CrI: 0.01–0.31]). Insufficient data were available to ascertain if age altered the effects of BA supplementation, but subset analyses were conducted to investigate the impact of sex and the method used to measure MCarn, using effect sizes generated from supplementation groups only. Sixteen studies were selected that used the most common dosing protocol (cumulative dose between 130 and 180 g) comprising a total of 56 effect sizes. For the sex comparison there were eight effect sizes from a female only group, 38 effect sizes from a male only group and 10 effect sizes from a mixed group. No substantive evidence of an effect of sex was obtained (male vs. female: −0.32 [95%CrI: −1.1–0.43]; male vs. mixed: −0.00 [95%CrI: −0.95–0.88]). Across the 16 studies, 40 effect sizes were obtained from MCarn values measured with non-invasive scanning devices (i.e., HR-MRS) and 16 effect sizes obtained with muscle biopsy based analyses (mainly assessed by HPLC, with one study using UPLC and one using mass spectrometry), with some evidence of increased effects with HPLC (0.16 [95%CrI: 0.01–0.43]).

### Emax Model

**Figure 4:**
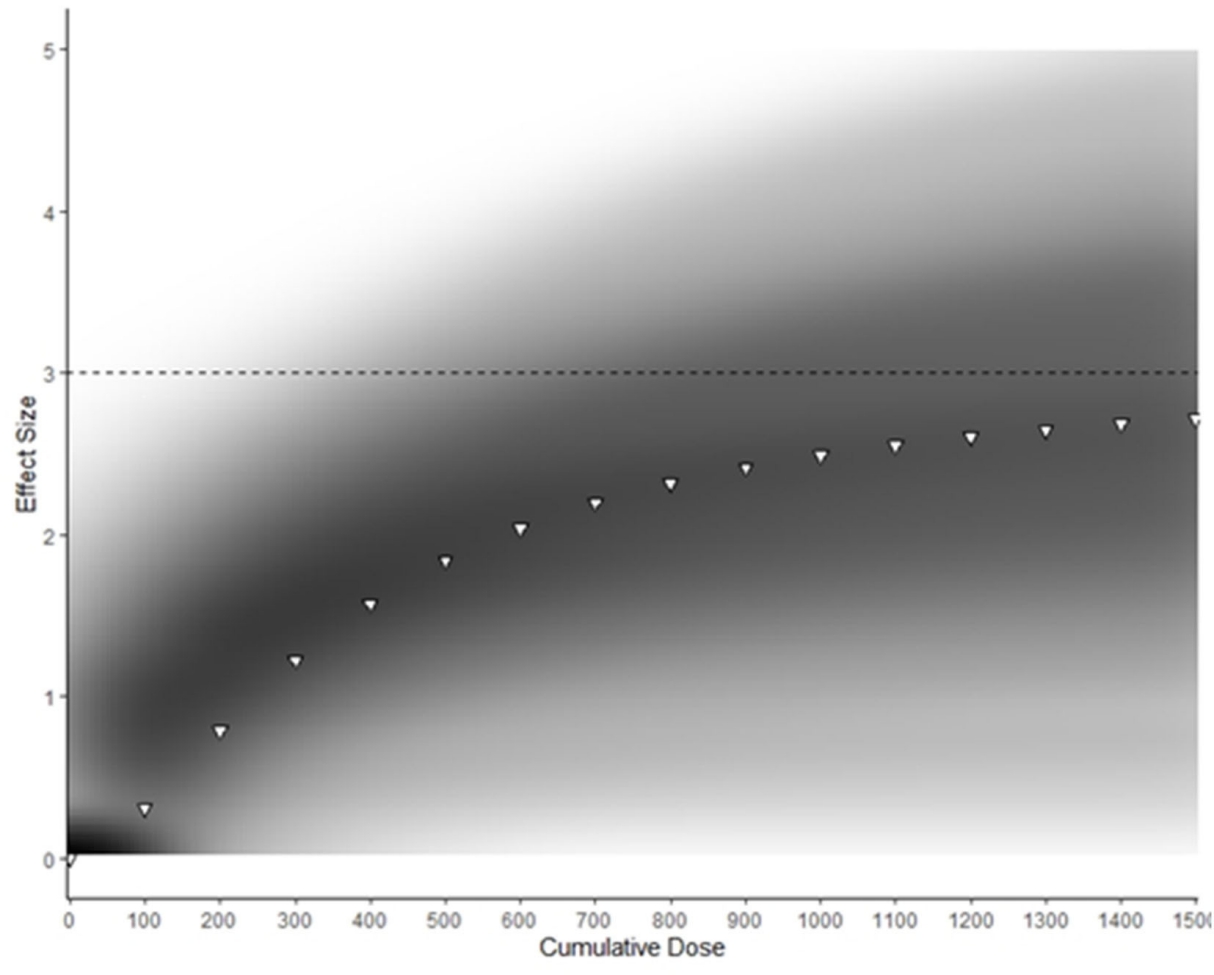
Density plot of Bayesian Emax model predicting effect of cumulative BA supplementation on muscle carnosine content. Darker areas represent more common Emax trajectories. White triangles represent Emax generated with median parameter values. The dotted line represents the predicted maximum effect of BA supplementation on MCarn.

The predicted maximum effect of BA supplementation (Emax) was 3.0 [50%CrI: 2.2–3.7] and the estimated total cumulative dose (*g*) required to achieve 50% of this maximum effect (ED50) was 377 g [50%CrI: 210–494]. A density plot with the Emax curve generated from median parameter values is provided in Figure 4. An extrapolation of posterior samples from the Emax model was performed to estimate probabilities that percentage of maximum effect could be achieved with cumulative doses ranging from 1,000 to 1,500 g (see Table 2). These results estimated, for example, that the probability of obtaining at least 70% of maximum effect with a cumulative dose of 1,000 g was 0.68.

**Table 2:** Probability table representing the chance that various cumulative doses (columns) create a response greater than the specified percentage of EMax (rows) based on Bayesian model generated.

**% EMax**	**1,000 g**	**1,100 g**	**1,200 g**	**1,300 g**	**1,400 g**	**1,500 g**
70%	0.68	0.73	0.77	0.80	0.83	0.85
80%	0.45	0.48	0.51	0.54	0.56	0.59
90%	0.31	0.33	0.35	0.37	0.38	0.40

*Values in table represent probabilities (p) 0 ≤ p ≤ 1*.

## Discussion

The purpose of this study was to conduct a comprehensive analysis with various modeling techniques to synthesize existing knowledge about the MCarn response to BA supplementation. Collectively, our findings, based on all models employed, indicated that human skeletal muscle has large capacity for MCarn accumulation, and that commonly used protocols (e.g., 4 weeks at 6.4 g·day^−1^) may not come close to saturating MCarn. Baseline values do not appear to influence subsequent response to supplementation and the non-linear response to supplementation was not influenced by sex. Analysis of individual data indicate that MCarn is relatively stable in the absence of intervention, and that effectually all (99.3% [95%CrI: 96.2–100]) participants respond to BA supplementation.

Our analyses indicate humans have large capacity for non-linear MCarn accumulation in response to BA supplementation. Figure 4 shows that BA supplementation can lead to a maximum effect size of ~3. Take, for example, the individual data set used in the current analysis, which had a baseline mean ± SD MCarn of 22.9 ± 8.7 mmol·kgDM^−1^. Intake of 1,500 g of BA is estimated to lead to an approximate increase of three times this standard deviation, (i.e., ~26.1 mmol·kgDM^−1^). It is important to highlight that these estimates are based on the median expected effect, and considerable inter-individual variation is likely. Additionally, estimates at the higher end of the curve described in Figure 4 should be interpreted with caution, as a paucity of data based on very high doses limits precision regarding the point at which human skeletal muscle saturation occurs. Despite these caveats, our data provides new insight into the nature of the MCarn response to BA supplementation, and how this differs to other commonly used dietary supplements, such as creatine. Human skeletal muscle appears to reach creatine saturation at ~140–160 mmol·kgDM^−1^ (Harris et al., 1992) and this can be achieved within 5 days of high-dose supplementation. Response to creatine supplementation is largest in those with lowest baseline levels, whereas individuals whose creatine content is habitually closer to this saturation point gain smaller benefit from supplementation (Harris et al., 1992). In contrast, we observed no evidence that baseline MCarn influenced response to supplementation. This makes sense when considered in relation to our predictive model, as it seems that humans have large capacity to accumulate MCarn—far greater than is achieved with commonly used protocols (e.g., 179.2 grams provided as 6.4 g·day^−1^ for 4 weeks). This may be because baseline MCarn contents (~25 mmol·kgDM^−1^) are substantially lower than predicted maximum capacity, whereas humans seem to habitually maintain creatine content at levels far closer to the proposed creatine saturation limit of ~140–160 mmol·kgDM^−1^.

Our model indicates that MCarn increase in response to BA supplementation is non-linear, and that the greatest increases occur in the earlier stages of supplementation. This finding aligns with a recent theoretical model proposed by Spelnikov and Harris (2019), which describes absolute MCarn increases as a product of both synthesis and decay, with carnosine synthesis considered to be constant in relation to time and first order to daily BA dose. Similarly, carnosine decay is also considered to be first order, but to relate to total MCarn content. As such, carnosine decay increases when absolute content is higher and so the rate of MCarn accumulation due to BA induced elevations in synthesis will slow, as illustrated in Figure 4. Tissue saturation represents the point at which the rates of synthesis match decay, and so content remains constant despite continued supplementation. The exact point, and nature, of this saturation point is not currently known. Does human skeletal muscle have a largely uniform saturation point, after which no further increases can be attained (as seems to be the case with creatine)? Or does capacity to accumulate MCarn vary widely between individuals, with each having their own upper limit? Currently, insufficient data using very high BA protocols on MCarn precludes the answering of this question, but one thing that is clear is that human skeletal muscle has large capacity to uptake BA and to increase MCarn, and that in the absence of intervention, MCarn is maintained at levels far below its maximal capacity.

The Emax model illustrated in Figure 4 clearly shows that very large amounts of BA are required to reach MCarn saturation. Theoretically, the greater the increase in MCarn content, the greater its ability to buffer, and to contribute to other processes such as anti-oxidation and anti-glycation, and so intuitively, attaining the largest increases possible seems desirable. But evidence on this hypothesis is conflicting. Two individual studies reported that larger MCarn increases were associated with greater performance effects (Hill et al., 2007; Saunders et al., 2017b), but this assertion is not supported by meta-analytic data, which indicates that the total dose ingested does not influence its effect on exercise performance (Saunders et al., 2017a). It would be counterintuitive to believe that performance benefits could linearly increase with ever-increasing MCarn, given that numerous factors, apart from acidosis, contribute to fatigue, and so it makes sense that at some point, performance benefits must plateau. Identification of the lowest MCarn increase necessary to elicit an ergogenic effect, along with the point after which no further benefits can be obtained would have large potential to enhance the applicability and efficacy of BA supplementation strategies. For example, it seems that the largest gains in MCarn are attained in the earlier phases of supplementation (see Figure 4). It would be of interest to identify if strategies such as meal co-ingestion (Stegen et al., 2013), intake in proximity to training (Bex et al., 2015), or intake in slow-release capsules (Varanoske et al., 2018) can influence the early response to supplementation (Perim et al., 2019) and whether this, in turn, meaningfully impacts exercise performance.

In addition to investigating whether or not greater MCarn increases are likely to bring about greater benefits, it is also important to weigh up the potential cons, against the potential pros, of this approach. From a practical point of view, dosing protocols of the magnitude required to cause saturation would be challenging. Additionally, BA supplementation in its current doses is regarded as having no adverse effects (Dolan et al., 2019b), but it is unknown if this will remain true at the substantially higher doses that are apparently required to reach saturation. Paresthesia, which is commonly described as a “pricking” or “tingling” sensation, commonly occurs during BA supplementation, likely due to the binding of BA to the peripheral neuronal receptor MrgprD (Liu et al., 2012). This sensation is not considered to be harmful but may be deemed unpleasant by some individuals. Paresthesia intensity is related to the timing of peak blood BA concentrations (Harris et al., 2006) and it is possible that large dosing increases, of the magnitude predicted to be necessary to achieve MCarn saturation, may invoke sensations deemed intolerable. Another theoretical adverse effect of prolonged BA supplementation is a decrease in taurine content, given that the two share a transporter (Tau-T) (Shaffer and Kocsis, 1981). We have previously reported that very high BA doses (namely those commonly used in animal trials) result in a substantial depletion of intracellular taurine (Dolan et al., 2019b) but the same does not hold true for human studies (Dolan et al., 2019b; Saunders et al., 2020), likely due to the substantially lower doses typically employed (Dolan et al., 2019b). It is possible that the very high doses apparently required for MCarn saturation, may lead to taurine reductions, and so some caution must be taken in attempting to implement substantially higher doses than those currently in use. Similarly, previous research highlighted that L-histidine is also required for carnosine synthesis, and that chronic BA supplementation may cause depletion of the free histidine pool, which in itself may have implications given the wide range of physiological processes that histidine contributes to Blancquaert et al. (2017). Similar to that which was observed for taurine, meta-analytic data indicated that BA dosing protocols within the ranges commonly used do not impact the free histidine pool (Dolan et al., 2019b), however no evidence currently exists to indicate whether or not this would remain true in the event of substantially increased BA dosing protocols. Collectively, the available evidence indicates that achieving the very high MCarn levels that the current Emax model indicates are possible, but may not be desirable, due to practical and safety issues. We suggest that in lieu of investigating means of maximizing intracellular carnosine content, future research efforts should instead focus on the point at which maximum ergogenic benefits are attained, as well as the point after which no further ergogenicity occurs.

The current analysis also brought to light some interesting points about the nature of the MCarn response to supplementation, which has implications for future study design. In the absence of intervention, MCarn seems to be relatively stable, likely due to low intramuscular carnosinase and roughly equivalent synthesis and degradation rates (Boldyrev et al., 2013). Our analysis of individual data indicated typical variation of ~4 mmol·kgDM^−1^ across a 4-weeks period. Reliability data indicate that two muscle samples taken from the same biopsy cut vary by ~1 mmol·kgDM^−1^, and so measurement error likely accounts for at least a quarter of this variation, and probably a lot more given that this estimate was based on samples taken moments apart and from the same biopsy cut. Interestingly, both within and between study variance were large and similar. A large proportion of this sampling error is likely due to small sample sizes. Typically, the use of a control group would be recommended to normalize the effects of the intervention against those of usual biological variability (Swinton et al., 2018). But in this situation, we observed little variation in placebo group MCarn, while the effects of intervention studies when analyzed both with, and without, controlling for the effects of the placebo group were similar (ES [95%CrI]: 1.7 [1.3–2.1] vs. 1.5 [1.2–1.8]). This implies that the control group adds little value to the analysis, likely because of MCarn stability and the large effect of supplementation. In future investigations of the MCarn response to BA in young healthy males (and particularly those for which resources are limited) it may be prudent to direct resources toward the intervention group, in order to reduce within study variance. It is important to note that this recommendation applies only to studies on the MCarn response to BA supplementation. The influence of BA supplementation on exercise performance, or clinical outcomes, is far less well-characterized and subject to substantially more sources of internal and external variability and so control groups are essential in studies for which exercise, or clinical effectiveness, is the primary outcome of interest.

In addition to characterizing the nature of MCarn response to BA supplementation, we also considered the influence of various potential moderators on this response. In relation to the method of assessment, it seems that lower effect estimates are generally observed when MCarn is measured using the H-MRS technique when compared to those obtained using HPLC analysis of muscle biopsies. Only one study showed no MCarn increase, despite using a commonly used dosing protocol of 6.4 g·day^−1^ for 28 days (Black et al., 2018). It is important to highlight that the MRS measurements reported in that study used a 1.5T magnet, as opposed to all others which used a 3T magnet. Given the incongruency of this finding in comparison to all others, it seems plausible that this may have occurred due to methodological inadequacies. When considering the influence of non-modifiable factors on the MCarn response to supplementation (namely age and sex), we could not conduct analyses on the influence of age, as insufficient data in older groups, and no data on younger groups, were available. Further research investigating the influence of BA supplementation on MCarn in older adults, along with potential therapeutic or ergogenic benefits, would be of interest, although it is worth highlighting that the one study that investigated a group aged 60–80 years did show comparable increases to other studies conducted in younger populations (del Favero et al., 2012). Women have previously been reported to have lower MCarn than men (Mannion et al., 1992; Everaert et al., 2011), which may be due to factors such as gender dimorphism in sex steroid concentrations (Peñafiel et al., 2004) or to variation in fiber-type composition (Dunnett et al., 1997; Hill et al., 2007; Painelli et al., 2018). Despite these differences, our data indicate that both men and women have a similar response to BA supplementation, indicating that the lower values previously reported in women are unlikely to relate to an inherent gender dysmorphism in the biological factors that underpin carnosine metabolism.

In conclusion, our findings indicate that human skeletal muscle has large capacity to accumulate carnosine. MCarn remains stable in the absence of intervention and neither low baseline MCarn levels, nor sex, influence the subsequent response to BA supplementation. In turn, these findings lead to other questions, the response to which may have large implications for future practice. From the point of view of athletic performance, key questions include: what is the absolute MCarn increase required to elicit an ergogenic effect, along with the point after which no further benefits are attained? It is clear that 4 weeks of BA supplementation can be ergogenic, but can this be achieved earlier? Can strategies to enhance the early response to BA supplementation meaningfully impact the subsequent ergogenic benefits? The response to these questions may progress practical application of this supplementation strategy, with potential benefit to many athletic and clinical populations.

## Data Availability Statement

All datasets generated for this study are included in the article/Supplementary Material. Any additional information is available from the corresponding author upon reasonable request.

## Author Contributions

ED, PS, BS, and BG designed the research. ED and NR conducted the searches. NR, LO, and RS extracted all data. KN, RS, GY, BS, and VE collected all original data used in the individual analysis. ED and NR wrote the manuscript, with ongoing critical input from PS, BG, GA, and BS. All authors read and approved the final manuscript.

## Conflict of Interest

BS has previously received a scholarship from Natural Alternatives International (NAI), San Marcos, California for a study unrelated to this one. NAI has also partially supported an original study conducted within our laboratory. This company has not had any input (financial, intellectual, or otherwise) into this review. The remaining authors declare that the research was conducted in the absence of any commercial or financial relationships that could be construed as a potential conflict of interest.
