# Supplementary material for: The Muscle Carnosine Response to Beta-Alanine Supplementation: A Systematic Review With Bayesian Individual and Aggregate Data E-Max Model and Meta-Analysis
Source: Front Physiol. 2020 Aug 14;11:913. doi: 10.3389/fphys.2020.00913 (PMC7456894; doi:10.3389/fphys.2020.00913)
Supplement: Supplementary file 1 [file Table_1.DOCX]

**The Muscle Carnosine Response to Beta-Alanine Supplementation: A Systematic Review with Bayesian Individual and Aggregate Data E-Max Model and Meta-Analysis**

**Online Supplemental Material**

**Supplemental Table 1:** Overview of studies included in the meta-analysis

| **Author (date)** | **Population** | **N (PLA/BA)** | **Dosing Strategy / TCD (g)** | **MCarn measurement (unit)** | **Device** | **Pre MCarn (PLA)** | **Post MCarn (PLA)** | **Pre MCarn (BA)** | **Post MCarn (BA)** |
| --- | --- | --- | --- | --- | --- | --- | --- | --- | --- |
| Baguet et al. (1) | Recreationally trained men | 7/8 | 2.4g for 2 days (4.8g)  3.6g for 2 days (7.2g)  4.8g for 42 days (201.6g)  (Total = 213.6g) | SOL  TA  GAS | MRS3 | 5.85 ± 0.76  5.51 ± 0.62  6.98 ± 1.20 | 5.89 ± 0.91  4.55 ± 1.25  7.18 ± 1.38 | 5.63 ± 0.94  6.25 ± 1.11  7.66 ± 1.37 | 7.83 ± 1.74  7.93 ± 1.70  9.45 ± 1.78 |
| Baguet et al. (2) | Trained men and women rowers) | 9/8 | 5g for 49 days (245g) | SOL  GAS | MRS3 | 3.45 ± 0.62  4.87 ± 1.07 | 3.29 ± 0.57  4.69 ± 1.30 | 3.13 ± 0.58  4.57 ± 0.56 | 4.48 ± 1.33  5.86 ± 1.63 |
| Bex et al. (3) | Trained men (road cyclists, swimmers, flat-water kayakers) | 0/35 | 6.4g for 23 days (147.2g) | SOL  GAS  DEL | MRS3 | - | - | 0.14 ± 0.04  0.16 ± 0.04  0.14 ± 0.03 | 0.21 ± 0.04  0.22 ± 0.03  0.22 ± 0.03 |
| Bex et al. (4) | Trained and sedentary men (jogging and cycling) | 0/28 | 6.4g for 23 days (147.2g) | SOL  GAS | MRS3 | - | - | 5.00 ± 0.86  8.33 ± 1.35 | 7.83 ± 1.38  11.38 ± 2.33 |
| Black et al. (5) | Recreationally trained men | 10/10 | 6.4g for 42 days (268.8g) | VM  VL  RF  WT | MRS1.5 | 0.21 ± 0.10  0.24 ± 0.08  0.18 ± 0.07  0.23 ± 0.13 | 0.20 ± 0.07  0.29 ± 0.15  0.17 ± 0.06  0.23 ± 0.10 | 0.18 ± 0.05  0.21 ± 0.07  0.15 ± 0.12  0.20 ± 0.08 | 0.16 ± 0.05  0.21 ± 0.09  0.15 ± 0.07  0.19 ± 0.08 |
| Blancquert et al. (6) | Recreationally trained men and women | 0/20 | 6g for 23 days (138.0g) | SOL  GAS  VL | MRS3 | - | - | 4.56 ± 0.74  7.31 ± 1.11  5.06 ± 1.47 | 6.91 ± 0.78  9.51 ± 1.89  6.54 ± 0.96 |
| Carvalho et al. (7) | Trained men (cyclists) | 14/14 | 6.4g for 28 days (179.2g) | VL | HPLC/MS | 1.08 ± 0.62 | 1.21 ± 0.78 | 1.18 ± 0.51 | 1.85 ± 0.75 |
| Chung et al. (8) | Trained men (cyclists, triathletes) | 13/14 | 6.4g for 42 days (268.8g) | SOL  GAS | MRS3 | 3.77 ± 1.43  5.56 ± 1.76 | 4.30 ± 1.55  6.55 ± 1.11 | 3.95 ± 2.56  5.56 ± 1.55 | 9.41 ± 1.86  11.70 ± 1.92 |
| Church et al. (9) | Recreationally trained men and women | 20/10 | 6g for 28 days (168g)  12g for 14 days (168g)  (Total = 336g) | VL | HPLC | 8.24 ± 1.63 | 7.55 ± 2.06 | 8.06 ± 3.60  6.87 ± 2.14 | 12.22 ± 6.19  9.68 ± 3.06 |
| Cochran et al. (10) | Recreationally trained men | 12/12 | 3.2g for 70 days (224g) | VL | UPLC | 6.40 ± 1.80 | 6.20 ± 1.70 | 6.40 ± 1.30 | 9.70 ± 1.60 |
| Da Eira Silva (11) | Recreationally trained men | 0/14 | 6.4g for 28 days (179.2g) | GAS | HPLC | - | - | 22.17 ± 10.10 | 34.67 ± 12.85 |
| Danaher et al. (12) | Recreationally trained men | 5/3 | 4.8g for 28 days (134.4g)  6.4g for 14 days (89.6g)  (Total = 224g) | SOL  GAS | MRS3 | 5.94 ± 0.56  8.73 ± 1.08 | 6.33 ± 0.89  7.42 ± 0.74 | 5.57 ± 0.25  8.08 ± 0.68 | 10.48 ± 1.35  13.11 ± 1.97 |
| Del Favero et al. (13) | Sedentary men and women | 6/12 | 3.2g for 84 days (268.8g) | GAS | MRS3 | 1.35 ± 0.41 | 1.41 ± 0.38 | 1.20 ± 0.73 | 2.02 ± 0.85 |
| Derave et al. (14) | Trained men (track-in-field athletes) | 7/8 | 2.4g for 4 days (9.6g)  3.6g for 4 days (14.4g)  4.8g for 20 days (96g)  (Total = 120g) | SOL  GAS | MRS3 | 7.25 ± 1.47  8.56 ± 1.88 | 7.85 ± 1.04  9.99 ± 1.31 | 7.76 ± 1.36  10.16 ± 1.91 | 11.39 ± 1.38  13.90 ± 2.60 |
| Gross et al. (15) | Trained men (team sports, cycling, running, triathlon) | 9/8 | 3.2g for 38 days (121.6g) | TA  GAS  VI  VL | MRS3 | 6.80 ± 1.10  9.60 ± 1.20  5.70 ± 1.30  7.10 ± 0.70 | 7.10 ± 0.90  9.20 ± 1.60  6.10 ± 1.00  7.20 ± 1.00 | 6.90 ± 0.80  8.80 ± 1.00  5.60 ± 1.10  7.40 ± 1.60 | 9.40 ± 0.80  10.80 ± 1.10  8.20 ± 1.10  9.20 ± 1.40 |
| Harris et al. (16) | Recreationally trained men | 6/10 | 3.2g for 28 days (89.6g)  5.2g for 28 days (145.6g)  (Total = 235.2g) | VL | HPLC | 23.63 ± 5.95 | 25.49 ± 4.97 | 19.58 ± 3.71  24.23 ± 5.28 | 27.38 ± 2.97  35.37 ± 6.17 |
| Harris et al. (17) | Sedentary men | 0/7 | 3.2g – 28 days (89.6g) | VL | HPLC | - | - | 25.90 ± 4.30 | 41.30 ± 5.50 |
| Hill et al. (18) | Recreationally trained men | 6/6 | 4g for 7 days (28g)  4.8g for 7 days (33.6g)  5.6g for 7 days (39.2g)  6.4g for 56 days (358.4g)  (Total = 459.2g) | VL | HPLC | 23.60 ± 2.40 | 23.90 ± 2.50 | 19.90 ± 1.90 | 34.70 ± 3.70 |
| Kendrick et al. (19) | Recreationally trained men | 13/13 | 6.4g for 28 days (179.2g) | VL | HPLC | 29.17 ± 9.82 | 27.29 ± 9.52 | 23.96 ± 5.94 | 36.77 ± 8.26 |
| Kendrick et al. (20) | Recreationally trained men | 7/7 | 6.4g for 28 days (179.2g) | VL | HPLC | 22.60 ± 2.10  24.20 ± 3.90 | 24.70 ± 3.70  23.40 ± 3.40 | 21.60 ± 7.80  25.20 ± 3.90 | 31.30 ± 6.90  31.80 ± 5.70 |
| Kresta et al. (21) | Trained women (running, cycling, swimming, resistance training, fitness classes) | 7/8 | 6.1g for 28 days (170.8g) | VL | HPLC | 15.70 ± 4.70 | 16.53 ± 4.80 | 19.74 ± 8.69 | 23.68 ± 1.56 |
| Saunders et al. (22) | Trained men (running, cycling, team sports) | 9/15 | 6.4g for 168 days (1075.2g) | VL | HPLC | 23.18 ± 5.90 | 23.46 ± 3.70 | 22.38 ± 4.46 | 42.52 ± 9.11 |
| Stegen et al. (23) | Sedentary men and women | 0/34 | 3.2g for 46 days (147.2g) | SOL  GAS | MSR3 | - | - | 3.46 ± 0.70  4.54 ± 1.08 | 5.21 ± 0.83  6.25 ± 1.16 |
| Stellingwerf et al. (24) | Sedentary men | 10/21 | 3.2g for 28 days (89.6g)  1.6g for 28 days (44.8g)  1.6g for 56 days (89.6g)  (Total = 224g) | GAS  TA | MRS3 | 9.09 ± 0.51  6.01 ± 0.41 | 9.05 ± 0.50  5.97 ± 0.45 | 8.88 ± 0.44  5.69 ± 0.22 | 10.77 ± 0.62  7.84 ± 0.43 |
| Varanoske et al. (25) | Sedentary men and women | 8/12 | 6g for 28 days (168g) | VL | HPLC | 7.28 ± 2.19 | 7.35 ± 2.29 | 7.28 ± 2.19 | 10.72 ± 3.67 |
| Varanoske et al. (26) | Recreationally trained men and women | 8/21 | 6g for 28 days (168g) | VL | HPLC | 8.12 ± 1.59 | 6.96 ± 3.61 | 7.38 ± 2.35 | 11.08 ± 2.64 |

SOL = Soleus; GAS = Gastrocnemius; TA = Tibialis Anterior; VI = Vastus Intermedius; VL = Vastus Lateralis; VM = Vastus Medialis; DEL = Deltoid; RF = Rectus Femoris; WT = Whole Thigh; MRS1.5 = Proton Magnetic Resonance Spectroscopy 1.5-T; MSR3 = Proton Magnetic Resonance Spectroscopy 3-T; HPLC/MS = High-Performance Liquid Chromatography Coupled to Electrospray Ionization Tandem Mass Spectrometry; HPLC = High-Performance Liquid Chromatography; UPLC = Ultra-performance Liquid Chromatography

**References:**

**(**1) Baguet A, Reyngoudt H, Pottier A et al. Carnosine loading and washout in human skeletal muscles. *J Appl Physiol*. 2009;106(3):837–42.

(2) Baguet A, Bourgois J, Vanhee L et al. Important role of muscle carnosine in rowing performance. *J Appl Physiol*. 2010;109(4):1096–101.

(3) Bex T, Chung W, Baguet A et al. Muscle carnosine loading by beta-alanine supplementation is more pronounced in trained vs. untrained muscles. *J Appl Physiol*. 2014;116(2):204–9.

(4) Bex T, Chung W, Baguet A et al. Exercise training and Beta-alanine-induced muscle carnosine loading. *Front Nutr*. 2015;7(2):13.

(5) Black MI, Jones AM, Morgan PT et al. The effects of β-alanine supplementation on muscle pH and the power-duration relationship during high-intensity exercise. *Front Physiol*. 2018;9(FEB):1–13.

(6) Blancquaert L, Everaert I, Missinne M et al. Effects of histidine and β-alanine supplementation on human muscle carnosine storage. *Med Sci Sport Exerc*. 2017;49(3):602–9.

(7) Carvalho VH, Oliveira AHS, de Oliveira LF et al. Exercise and β-alanine supplementation on carnosine-acrolein adduct in skeletal muscle. *Redox Biol*. 2018;18(July):222–8.

(8) Chung W, Baguet A, Bex T et al. Doubling of muscle carnosine concentration does not improve laboratory 1-Hr cycling time-trial performance. *Int J Sport Nutr Exerc Metab*. 2014;24(3):315–24.

(9) Church D, Hoffman J, Varanoske A et al. Comparison of two β-alanine dosing protocols on muscle carnosine elevations. *J Am Coll Nutr*. 2017;36(8):608–16.

(10) Cochran AJ, Percival ME, Thompson S et al. Beta-alanine supplementation does not augment the skeletal muscle adaptive response to six weeks of sprint interval training. *Int J Sport Nutr Exerc Metab*. 2015;25(6):541–9.

(11) Da Eira Silva V, De Salles Painelli V, Shinjo SK et al. Magnetic Resonance Spectroscopy as a Non-invasive Method to Quantify Muscle Carnosine in Humans: a Comprehensive Validity Assessment. *Sci Rep*. 2020;4908(10).

(12) Danaher J, Gerber T, Wellard RM et al. The effect of β-alanine and NaHCO3 co-ingestion on buffering capacity and exercise performance with high-intensity exercise in healthy males. *Eur J Appl Physiol*. 2014;114(8):1715–24.

(13) del Favero S, Roschel H, Solis MY et al. Beta-alanine (Carnosyn (TM)) supplementation in elderly subjects (60-80 years): effects on muscle carnosine content and physical capacity. *Amino Acids*. 2012;43(1):49–56.

(14) Derave W, Ozdemir MS, Harris RC et al. Beta-Alanine supplementation augments muscle carnosine content and attenuates fatigue during repeated isokinetic contraction bouts in trained sprinters. *J Appl Physiol*. 2007;103(5):1736–43.

(15) Gross M, Bieri K, Hoppeler H et al. Beta ‐ alanine supplementation improves jumping power and affects severe ‐ intensity performance in professional alpine skiers. 2014;05:1–23.

(16) Harris R, Tallon M, Dunnett M et al. The absorption of orally supplied beta-alanine and its effect on muscle carnosine synthesis in human vastus lateralis. *Amino Acids*. 2006;30(3):279–89.

(17) Harris R, Kim H, Kim C et al. Simultaneous changes in muscle carnosine and taurine during and following supplementation with β-Alanine. *Med Sci Sport Exerc*. 2010;42(S5):107.

(18) Hill C, Harris R, Kim H et al. Influence of β-alanine supplementation on skeletal muscle carnosine concentrations and high intensity cycling capacity. *Amino Acids*. 2007;32(2):225–33.

(19) Kendrick IP, Harris RC, Kim HJ et al. The effects of 10 weeks of resistance training combined with beta-alanine supplementation on whole body strength, force production, muscular endurance and body composition. *Amino Acids*. 2008;34(4):547–54.

(20) Kendrick IP, Kim HJ, Harris RC et al. The effect of 4 weeks beta-alanine supplementation and isokinetic training on carnosine concentrations in type I and II human skeletal muscle fibres. *Eur J Appl Physiol*. 2009;106(1):131–8.

(21) Kresta JY, Oliver J, Jagim A et al. Effects of 28 days of beta-alanine and creatine monohydrate supplementation on muscle carnosine, body composition and exercise performance in recreationally active females. *J Int Soc Sports Nutr*. 2014;11(1):55.

(22) Saunders B, De Salles Painelli V, De Oliveira LF et al. *Twenty-four weeks of β-alanine supplementation on carnosine content, related genes, and exercise*. 2017. 896–906 p.

(23) Stegen S, Blancquaert L, Everaert I et al. Meal and beta-alanine coingestion enhances muscle carnosine loading. *Med Sci Sports Exerc*. 2013;45(8):1478–85.

(24) Stellingwerff T, Anwander H, Egger A et al. Effect of two β-alanine dosing protocols on muscle carnosine synthesis and washout. *Amino Acids*. 2012;42(6):2461–72.

(25) Varanoske A, Hoffman J, Church D et al. β-Alanine supplementation elevates intramuscular carnosine content and attenuates fatigue in men and women similarly but does not change muscle l-histidine content. *Nutr Res*. 2017;48:16–25.

(26) Varanoske AN, Hoffman JR, Church DD et al. Comparison of sustained-release and rapid-release β-alanine formulations on changes in skeletal muscle carnosine and histidine content and isometric performance following a muscle-damaging protocol. *Amino Acids*. 2018;(0123456789):1–12.
